# Supplementary material for: ContScout: sensitive detection and removal of contamination from annotated genomes
Source: Nat Commun. 2024 Jan 31;15:936. doi: 10.1038/s41467-024-45024-5 (PMC10831095; doi:10.1038/s41467-024-45024-5)
Supplement: Supplementary file 1 — Supplementary Information [file 41467_2024_45024_MOESM1_ESM.pdf]

Supplementary Information for

ContScout: Sensitive detection and removal of contamination from annotated genomes

Balázs Bálint, Zsolt Merényi, Botond Hegedüs, Igor V. Grigoriev, Zhihao Hou, Csenge Földi, László G. Nagy

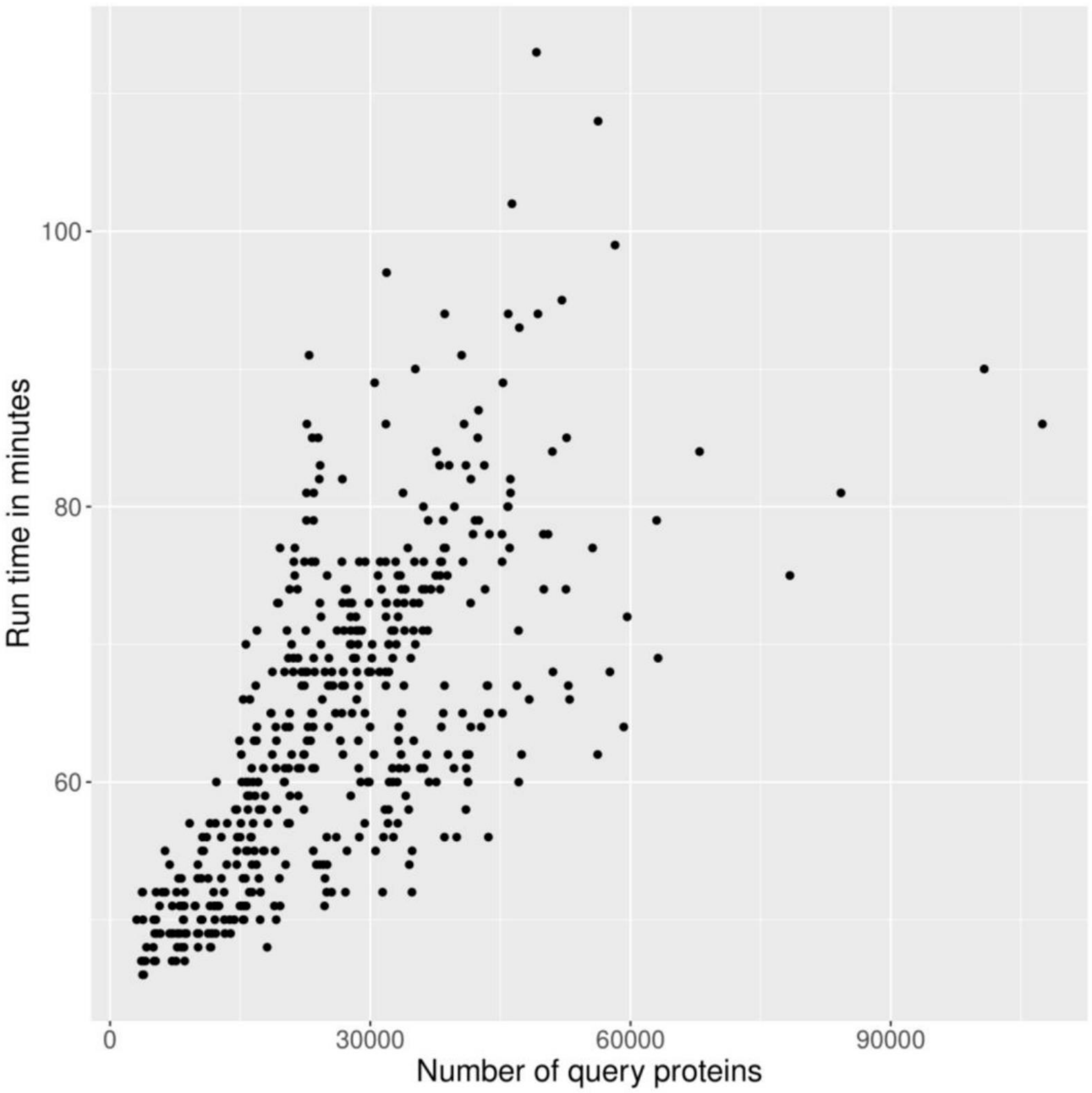

Supplementary Figure 1: ContScout run time statistics. Run time, measured as wall time minutes, were plotted as a function of query proteome size. Runs were performed using 24 CPU cores with the RAM usage being limited to 150 GB. Scatter plot contains 478 data points.

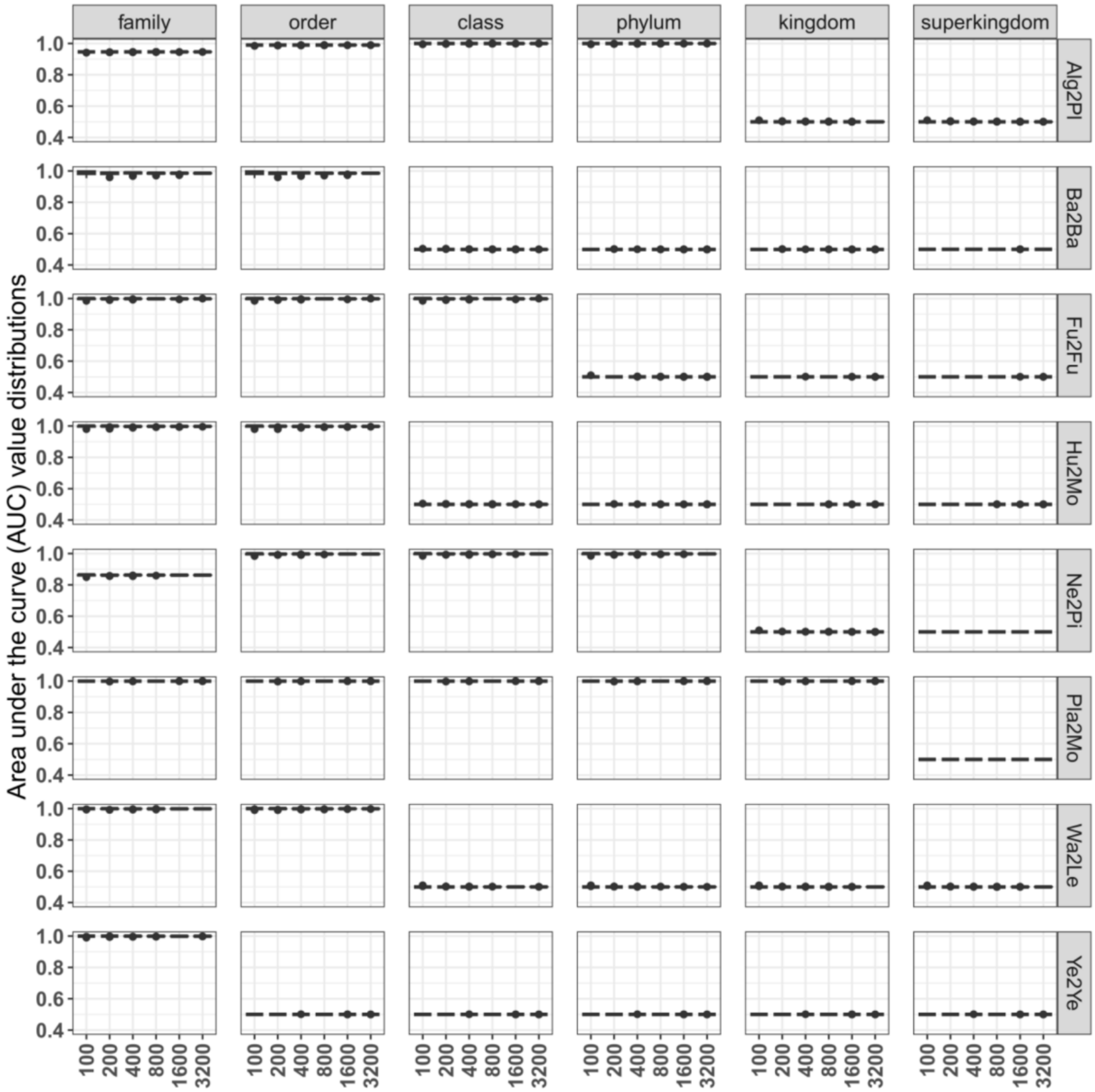

Supplementary Figure 2: ContScout performance assessment on directed synthetic mixes. A set of artificially contaminated genomes with source-recipient pairs mimicking biologically realistic contamination scenarios were generated. Matrix of box plots shows area under the curve (AUC) value distributions for the classification predictions made by ContScout, where column position of charts corresponds to the taxonomic rank at which decontamination was performed. Each row holds data from one directed source-recipient pair. Rows are labeled as follows: Alg2Pl=alga in plant, Ba2Ba=bacteria in bacteria, Fu2Fu=fungi in fungi, Ho2Mo=human in mouse, Ne2Pi=nematode in pig, Pla2Mo=Plasmodium in mosquito, Wa2Le=parasitic wasp in its moth host, Ye2ye=yeast in yeast. For more information on the mixed genomes see Supplementary Data 1. Within each of the boxplots, axis x refers to the amount of contamination proteins (100, 200, 400, 800, 1600 or 3200) that was spiked in the recipient genome. Each boxplot is based on 100 independent replications.

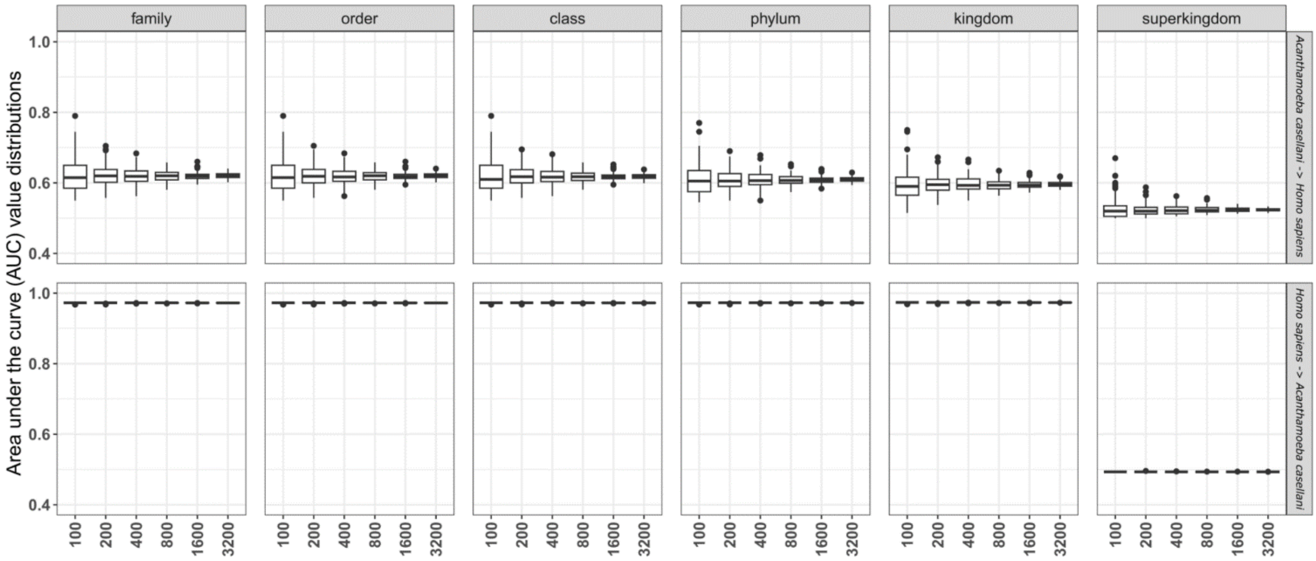

Supplementary Figure 3: example of a hard-to resolve case. Artificially contaminated mixtures were between *Acanthamoeba castellanii* and *Homo sapiens* genomes were generated representing both contaminant-recipient directions. Matrix of box plots shows area under the curve (AUC) value distributions for the classification predictions made by ContScout, where column position of charts corresponds to the taxonomic rank at which decontamination was performed. Data for the two separate contaminant-recipient directions are presented in separate rows. Within each of the boxplots, axis x refers to the amount of contamination proteins (100, 200, 400, 800, 1600 or 3200) that was spiked in the recipient genome.

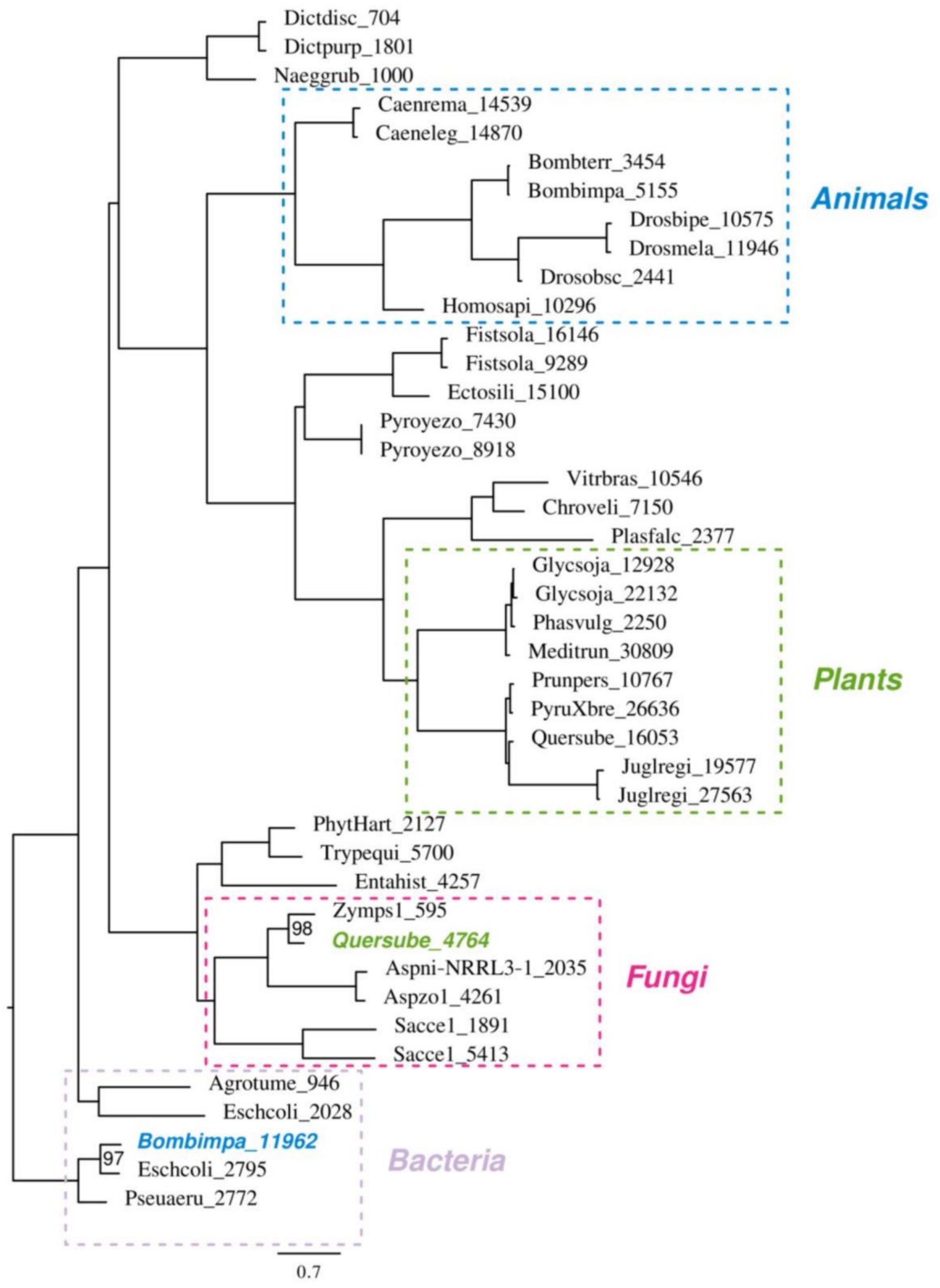

Supplementary Figure 4: Gene tree of the pyridoxal kinase protein family. Gene tree for of the ubiquitous pyridoxal kinase protein family has been inferred from the unfiltered 36-genome data set. Dotted lines around clades in the tree indicate major taxonomic groups, with animals being represented in blue, plants in green, fungi in magenta, and bacteria in pale purple. The *Quercus suber* protein Quersube_4764, that is positioned among fungal sequences, is labelled in green while *Bombus impatiens* protein Bombimpa_11962, that is most similar to bacterial sequences, is labelled in blue.

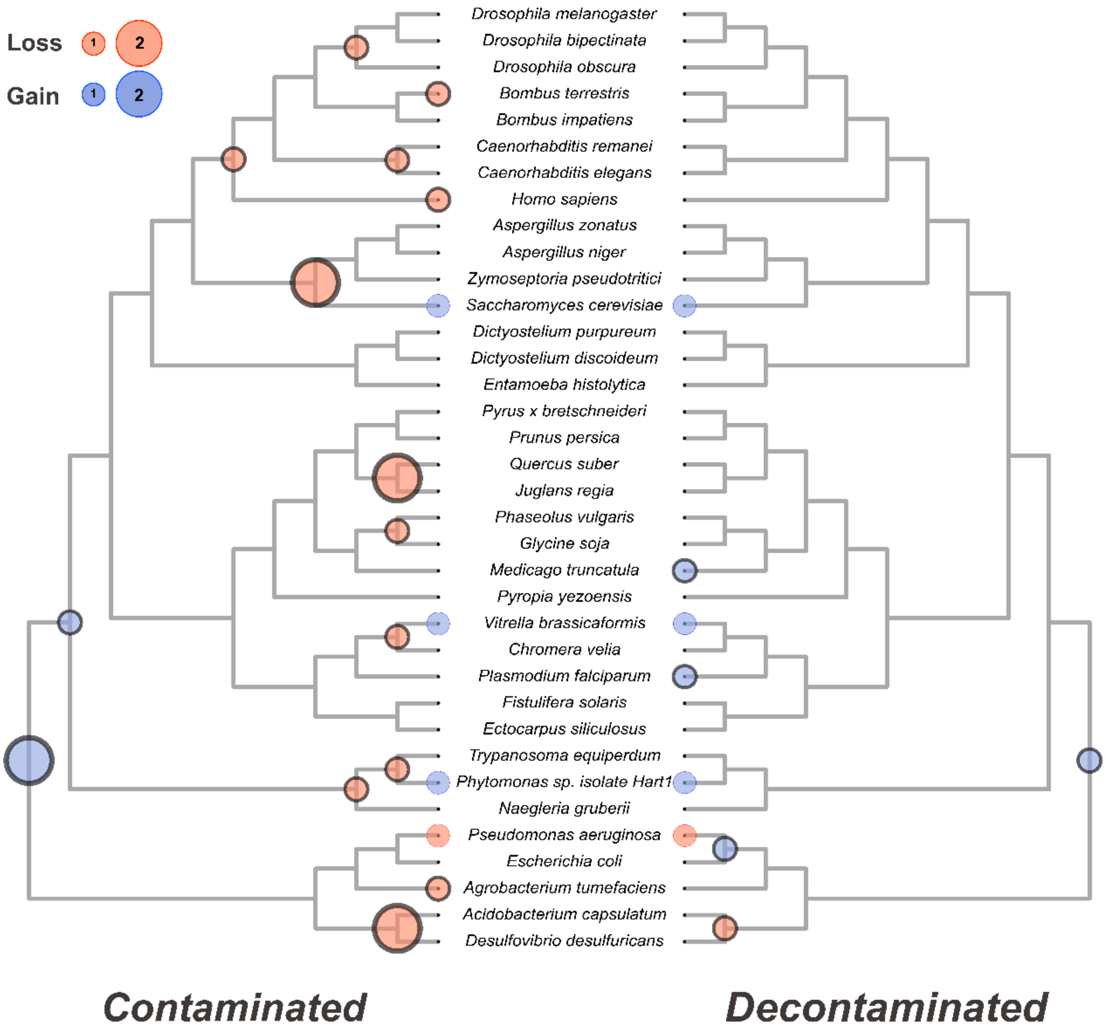

Supplementary Figure 5: Effect of contamination on the evolutionary history of the pyridoxal kinase family. Gene loss / gain events for the pyridoxal kinase family were inferred with COMPARE[1] from both the unfiltered and decontaminated 36-genomes data set and were mapped on the 3 species tree. Circle sizes are proportional to the number of events at internal nodes. Gene losses are represented by red circles while gains are indicated by blue circles Thick dark stroke lines around circles highlight loss / gain estimates that were affected by contamination. All changes that are shown in the figure are the consequence of two contaminating proteins within the family: Quersube_4764 and Bombimpa_11962.

Supplementary Table 1 Comparison of ContScout with FCS-GX. Abbreviations: TP=true positives, FP=false positives, KFC=known fungal contigs.

Short Name	Species	Lineage	FCS-GX only	ContScout only	Both
Aspzo1	*Aspergillus zonatus*	fungi	0	0	14 (TP:14)
Papixuth	*Papilio xuthus*	animal	19 (TP:19)	3 (TP:2, FP:1)	124 (TP:124)
Quersube	*Quercus suber*	plant	6	85	542 (KFC: 35/35)

Supplementary Table 2 Third-party software tools used for data manipulation, data analysis and visualization throughout the study.

Name	Version	URL
ape[2]	5.5	https://cran.r-project.org/web/packages/ape/index.html
BASTA[3]	1.4	https://github.com/timkahlke/BASTA
Biostrings**	2.62.0	https://bioconductor.org/packages/release/bioc/html/Biostrings.html
BUSCO[4]	5.4.4	https://busco.ezlab.org/
Compare[1]	v2023.03	https://github.com/zsmerenyi/compaRe
Conterminator[5]	570993be7f5f31ee357 183c9118bf3aa755758 70	https://github.com/steineggerlab/conterminator
ContScout*	V2023.09	https://github.com/h836472/ContScout/tree/NatComm
Diamond[6]	2.0.4	https://github.com/bbuchfink/diamond
FCS-GX[7]	v0.4.0-3-g8096f62	https://github.com/ncbi/fcs
GenomicRanges[8]	1.46.1	https://bioconductor.org/packages/release/bioc/html/GenomicRanges.html
ggplot2[9]	3.4.1	https://ggplot2.tidyverse.org/
hipMCL[10]	V2020 06	https://bitbucket.org/azadcse/hipmcl/src/master/
Interproscan[11]	V5.44.79.0	https://ftp.ebi.ac.uk/pub/software/unix/iprscan/5/
Mafft[12]	7.0.407	https://mafft.cbrc.jp/alignment/software/
MMSeqs2[13]	bb0a1b3569b9fe115f3 bf63e5ba1da234748d e23	https://github.com/soedinglab/MMseqs2
OrthoFinder[14]	V2.4.1	https://github.com/davidemms/OrthoFinder
pheatmap**	1.0.12	https://CRAN.R-project.org/package=pheatmap
phytools[15]	0.7-80	https://cran.r-project.org/web/packages/phytools/index.html
pROC[16]	1.18.0	https://cran.r-project.org/web/packages/pROC/
R[17]	4.1.0	https://cran.r-project.org/bin/
RAxML[18]	8.2.12	https://github.com/stamatak/standard-RAxML
RColorBrewer**	1.1-2	https://cran.r-project.org/web/packages/RColorBrewer/index.html
rtracklayer	1.54.0	https://bioconductor.org/packages/release/bioc/html/rtracklayer.html
TrimAl[19]	1.2rev59	http://trimal.cgenomics.org/
WriteXLS**	6.4.0	https://cran.r-project.org/web/packages/WriteXLS/index.html

* present work

** R package without any scientific publication

Supplementary References

1. Nagy, L. G. *et al.* Latent homology and convergent regulatory evolution underlies the repeated emergence of yeasts. *Nat Commun* **5**, 4471 (2014).
2. Paradis, E. & Schliep, K. ape 5.0: an environment for modern phylogenetics and evolutionary analyses in R. *Bioinformatics* **35**, 526–528 (2019).
3. Kahlke, T. & Ralph, P. J. BASTA – Taxonomic classification of sequences and sequence bins using last common ancestor estimations. *Methods Ecol Evol* **10**, 100–103 (2019).
4. Simão, F. A., Waterhouse, R. M., Ioannidis, P., Kriventseva, E. V & Zdobnov, E. M. BUSCO: assessing genome assembly and annotation completeness with single-copy orthologs. *Bioinformatics* **31**, 3210–3212 (2015).
5. Steinegger, M. & Salzberg, S. L. Terminating contamination: large-scale search identifies more than 2,000,000 contaminated entries in GenBank. *Genome Biol* **21**, 115 (2020).
6. Buchfink, B., Xie, C. & Huson, D. H. Fast and sensitive protein alignment using DIAMOND. *Nat Methods* **12**, 59–60 (2015).
7. Astashyn, A. *et al.* Rapid and sensitive detection of genome contamination at scale with FCS-GX. *bioRxiv* 2023.06.02.543519 (2023) doi:10.1101/2023.06.02.543519.
8. Lawrence, M. *et al.* Software for Computing and Annotating Genomic Ranges. *PLoS Comput Biol* **9**, e1003118 (2013).
9. Wickham, H. *ggplot2*. (Springer New York, 2009). doi:10.1007/978-0-387-98141-3.
10. Azad, A., Pavlopoulos, G. A., Ouzounis, C. A., Kyrpides, N. C. & Bulųc, A. HipMCL: a high-performance parallel implementation of the Markov clustering algorithm for large-scale networks. *Nucleic Acids Res* **46**, e33–e33 (2018).
11. Jones, P. *et al.* InterProScan 5: genome-scale protein function classification. *Bioinformatics* **30**, 1236–1240 (2014).
12. Katoh, K. & Standley, D. M. MAFFT Multiple Sequence Alignment Software Version 7: Improvements in Performance and Usability. *Mol Biol Evol* **30**, 772–780 (2013).
13. Steinegger, M. & Söding, J. MMseqs2 enables sensitive protein sequence searching for the analysis of massive data sets. *Nat Biotechnol* **35**, 1026–1028 (2017).
14. Emms, D. M. & Kelly, S. OrthoFinder: phylogenetic orthology inference for comparative genomics. *Genome Biol* **20**, (2019).
15. Revell, L. J. phytools 2.0: an updated R ecosystem for phylogenetic comparative methods (and other things). *PeerJ* **12**, e16505 (2024).
16. Robin, X. *et al.* pROC: an open-source package for R and S+ to analyze and compare ROC curves. *BMC Bioinformatics* **12**, 77 (2011).
17. R_Core_Team. R: A Language and Environment for Statistical Computing. Preprint at https://www.r-project.org (2022).
18. Stamatakis, A. RAxML version 8: a tool for phylogenetic analysis and post-analysis of large phylogenies. *Bioinformatics* **30**, (2014).
19. Capella-Gutierrez, S., Silla-Martinez, J. M. & Gabaldon, T. trimAl: a tool for automated alignment trimming in large-scale phylogenetic analyses. *Bioinformatics* **25**, 1972–1973 (2009).
