# Supplementary material for: ContScout: sensitive detection and removal of contamination from annotated genomes
Source: Nat Commun. 2024 Jan 31;15:936. doi: 10.1038/s41467-024-45024-5 (PMC10831095; doi:10.1038/s41467-024-45024-5)
Supplement: Supplementary file 4 — Description of Additional Supplementary Files [file 41467_2024_45024_MOESM4_ESM.pdf]

Description of Additional Supplementary Files

Supplementary Data 1
Description: Synthetic mixtures created to assess ContScout performance

File name: Supplementary Data 2
Description: Known bacterial HGT events tested screened with ContScout

File name: Supplementary Data 3
Description: Details of the 844 eukaryote genomes used in the study

Supplementary Data 4
Description: Proteins identified by ContScout as contamination from the 844-genomes data set

Supplementary Data 5
Description: High-level taxon summary of proteins removed by ContScout from the 200 most contaminated genomes

Supplementary Data 6
Description: Details of the 36 genomes used in the study to illustrate the effect of contaminations
